# Prevention of Sepsis in Children: A New Paradigm for Public Policy

**DOI:** 10.1155/2012/437139

**Published:** 2011-12-18

**Authors:** Carley Riley, Derek S. Wheeler

**Affiliations:** ^1^Division of Critical Care Medicine, Cincinnati Children's Hospital Medical Center, 3333 Burnet Avenue, Cincinnati, OH 45229-3039, USA; ^2^The James M. Anderson Center for Health Systems Excellence, Cincinnati Children's Hospital Medical Center, 3333 Burnet Avenue, Cincinnati, OH 45229-3039, USA; ^3^Department of Pediatrics, University of Cincinnati College of Medicine, 3333 Burnet Avenue, Cincinnati, OH 45229-3039, USA

## Abstract

Sepsis is one of the leading causes of death worldwide. While the management of critically ill patients with sepsis is certainly better now compared to 20 years ago, sepsis-associated mortality remains unacceptably high. Annual deaths from sepsis in both children and adults far surpass the number of deaths from acute myocardial infarction (AMI), stroke, or cancer. Given the substantial toll that sepsis takes worldwide, prevention of sepsis remains a global priority. Multiple effective prevention strategies exist. Antibiotic prophylaxis, immunizations, and healthcare quality improvement initiatives are important means through which we may reduce the morbidity and mortality from sepsis around the world. Inclusion of these strategies in a coordinated and thoughtful campaign to reduce the global burden of sepsis is necessary for the improvement of pediatric health worldwide.

## 1. Introduction

According to the National Center for Health Statistics and the Centers for Disease Control and Prevention, sepsis was the 10th leading cause of death in the United States overall in 2007 [1]. There are between 77 to 240 new cases of sepsis per 100,000 population each year [2, 3]. More importantly, several experts believe that the incidence of sepsis will continue to increase by approximately 1.5% every year, resulting in an additional 1 million cases per year by 2020 [2, 4, 5]. Several factors are believed to be responsible for this increase. The population is growing older, and patients are living longer, even in the face of diseases that were previously considered universally fatal. Hospitalized patients are becoming more dependent upon the use of invasive devices and technology, all of which are associated with increased risk of infection. In addition, the epidemiology of sepsis is changing as another consequence of the greater use of invasive devices and technology in hospitalized patients. Classically, these patients died from gram-negative sepsis. However, infections with gram-positive bacteria and *Candida* species are now becoming more prevalent. The number of cases of fungal sepsis, which is associated with markedly worse outcomes, has increased by more than 200 percent between 1979 and 2000 [2]. Clearly, fungal sepsis is becoming an increasingly important entity and deserves further attention.

The story in children is fairly similar. There are between 20,000 and 42,000 cases of severe sepsis every year in the United States alone, half of which occur in children with underlying diseases like cancer and congenital heart disease [6, 7]. Again, similar to the situation in adults, the incidence of sepsis in critically ill children is expected to increase as more children survive diseases that were previously considered uniformly fatal [8]. While studies on the changing epidemiology of sepsis are far from conclusive, the increased utilization of invasive devices and the longer survival rates from diseases such as cancer which were previously uniformly fatal will likely lead to an increased incidence of invasive *Candida* infections and other opportunistic infections.

While the management of critically ill patients with sepsis is certainly better now compared to 20 years ago [9–11], sepsis-associated mortality remains unacceptably high. Annual deaths from sepsis in both children and adults far surpass the number of deaths from acute myocardial infarction (AMI), stroke, or cancer [4]. Recent estimates suggest that there are approximately 4,500 children who die every year from sepsis in the United States alone [6, 12]. The actual number of deaths associated with sepsis is likely to be much higher, as many patients usually die from sepsis during the course of an underlying disease, such as prematurity, congenital heart disease, or cancer. In many of these cases, deaths are frequently attributed to the underlying disease process, rather than to sepsis [4, 6, 13, 14]. Therefore, the impact of sepsis both in terms of annual healthcare costs and attributable mortality is likely to be greatly underestimated.

Pediatric sepsis is a growing public health problem in the developing world as well. According to data from the World Health Organization (WHO), the United Nations Children's Fund (UNICEF), and the Bill and Melinda Gates Foundation, nearly 70% of the 8 million deaths in children <5 years of age were due to infectious diseases, such as malaria, dengue fever, pneumonia, influenza, and AIDS [15]. As sepsis is the final common pathway in most, if not all, of these diseases, sepsis can and should be considered the no. 1 killer of children worldwide!

Unfortunately, sepsis consistently receives markedly lower amounts of research dollars from the National Institutes of  Health compared to the other leading causes of death in the United States. Surprisingly, sepsis receives less research funding compared to smallpox [16], a disease which has been reportedly “wiped off the face of the Earth” [17]. Moreover, sepsis receives relatively little attention in the lay public—most of the public has never even heard of the term “sepsis” [18]. Unfortunately, knowledge about sepsis as a disease entity amongst health care workers is not much better [19–21]. A recent international survey of over 1,058 physicians from a wide variety of disciplines, including critical care medicine, found that nearly two-thirds of the physicians surveyed believed that a common definition for sepsis was lacking [19].

Several promising therapies in preclinical models of sepsis have universally failed to live up to initial expectations in subsequent clinical trials in both critically ill children and adults [11]. In fact, to date, there have been only three positive clinical trials in critically ill adults with sepsis–early goal-directed therapy (EGDT) [22], activated protein C (drotrecogin alfa, Xigris, Eli Lilly and Co, Indianapolis, IN) [23], and afelimomab, a monoclonal antibody F(ab')2 fragment directed against tumor necrosis factor (TNF)-*α* [24]. With the one possible exception of early goal-directed therapy [25–30], none of these therapies have proven successful in critically ill children. Moreover, Eli Lilly and Company recently announced that it was withdrawing drotrecogin alfa (Xigris) from the market after the drug failed to improve patient survival in a recently concluded prospective, placebo-controlled, multicenter trial in Europe (Prowess-Shock study) [31].

Given all of these factors, we believe that a new approach to pediatric sepsis research and public policy is necessary, perhaps modeled on the successful public health and policy campaign to reduce mortality from acute myocardial infarction (AMI), currently the leading cause of death in the United States [10]. The care of patients with AMI has benefited greatly from worldwide media attention, an unprecedented level of research funding, and extensive resources directed towards prevention and management, all of which have resulted in a greater quality of care and a subsequent reduction in AMI-related mortality. A similar approach can and should be directed towards improving the quality of care of critically ill patients with sepsis. Perhaps more importantly, however, any effort to reduce morbidity and mortality from sepsis around the globe must include the expanded use of prevention strategies. Multiple prevention strategies have already proven invaluable in decreasing the incidence of sepsis worldwide. Herein, we will highlight some of the prevention strategies with the best supportive evidence, including antibiotic prophylaxis, immunizations, and healthcare quality improvement initiatives. We believe that the first step towards the successful management of critically ill patients with sepsis is the primary prevention of sepsis itself.

## 2. Antibiotic Prophylaxis

Antibiotic prophylaxis has been instrumental in efforts to decrease the incidence of sepsis in multiple different settings. Prophylaxis reduces the risk of sepsis from group B *Streptococcus *in neonates, from encapsulated organisms in patients with asplenia, and from subacute bacterial endocarditis in individuals with structural heart disease (Table 1). Each of these interventions is vital for the prevention of overwhelming infection for its respective population. We will focus particularly on the prevention of invasive group B *Streptococcus* (GBS) infection as a powerful example of how antibiotic prophylaxis can prevent sepsis.

### 2.1. GBS: Preventing Sepsis with Antibiotic Prophylaxis

In the past two decades, intrapartum antibiotic chemoprophylaxis has substantially reduced the incidence of neonatal sepsis due to GBS, from nearly 2 cases per 1,000 live births to less than 0.5 cases per 1000 live births [32]. Prevention of neonatal GBS infection is important because the infection results in death and devastating morbidity for a substantial number of children worldwide. Early neonatal infection from GBS is vertically transmitted, often presents within the first hours of life and most commonly within the first forty-eight hours of life, and is characterized by rapid clinical deterioration [32]. During the early 1970s in the United States, the mortality rates from early neonatal GBS sepsis were 50% or higher with subsequent advances in neonatal medical care reducing the mortality rates to 4 to 6 percent [32]. Long-term morbidity from early neonatal GBS infection, however, includes some degree of neurologic deficit in approximately half of infected children and severe neurologic sequelae in nearly one-third of infected children [32]. Reduction in the burden of early neonatal GBS sepsis through antibiotic prophylaxis has therefore been a significant achievement.

The evolution of routine antibiotic prophylaxis for neonatal GBS required more than two decades of efforts by dedicated community of health professionals. In the United States, the medical community recognized GBS as the leading infectious cause of early neonatal morbidity and mortality in the 1970s [33]. Clinical research then revealed that use of intrapartum antibiotics in women at risk of transmitting GBS to their children prevented invasive infection in the first week of life [33]. In the 1990s, the American College of Obstetricians and Gynecologists (ACOG), the Centers for Disease Control and Prevention (CDC), and the American Academy of Pediatrics (AAP) recommended intrapartum prophylaxis for the prevention of early neonatal GBS disease [33].

The adoption of universal screening for maternal GBS at 35 to 37 weeks gestation followed by recommended antibiotic prophylaxis during labor to colonized women has translated to a substantial decline in the incidence of early neonatal GBS disease [33]. Prior to active prevention, incidence of neonatal GBS disease in the United States was approximately 7,500 cases per year [33]. Since the initiation of prevention through prophylaxis, the incidence of invasive early GBS disease has declined approximately by 80% [33]. Rates of clinical sepsis demonstrate a continuous decline throughout the 1990s and into the 2000s with a striking decline among term infants in the two years following the issuance of the 1997 GBS prevention guidelines [33]. Overall, in the United States, the incidence has declined from 1.7 cases per 1000 live births in the preprevention era to less than 0.4 cases per 1000 live births after the reissuance of guidelines in 2002 [32, 33].

Though antibiotic prophylaxis clearly prevents overwhelming infection, the medical community has needed to examine potential concerns around prophylaxis and its effects. One concern is that widespread use of antibiotics for prophylaxis may lead to the development of antibiotic resistance. With respect to prophylaxis for GBS, developing resistance has not been a problem. GBS continues to be susceptible to penicillin, ampicillin, and first-generation cephalosporins, though GBS isolates with increasing minimum inhibitory concentrations to penicillin and ampicillin have been observed [33].

Another concern has been that prophylactic antibiotic use may alter the presentation of illness if infection occurs in spite of prophylaxis. If this alteration delays recognition of the disease, it may interfere with the optimal management of infection. Fortunately, this has not occurred with neonatal GBS. Since the guidelines were published in 1996, multiple studies have demonstrated no significant difference in the clinical presentation of neonates with early GBS disease between those who have been exposed to intrapartum antibiotics and those who have not been exposed [33]. Though there are concerns to be aware of when using antibiotic prophylaxis, in the case of GBS, antibiotic prophylaxis has not created these undesired effects.

Nevertheless, antibiotic prophylaxis remains imperfect. For example, prophylaxis has not affected rates of maternal colonization with GBS. Despite decades of routine prophylaxis, colonization rates for GBS remain unchanged, which therefore means that risk of neonatal exposure to GBS remains unchanged [33]. Moreover, due to false negative results, universal screening fails to identify a percentage of pregnant women colonized with GBS and thereby fails to protect the children born to those women [33]. Though there are inherent shortcomings to prophylaxis as a prevention strategy, there also appear to be imperfections in how prophylaxis is realized. In the case of neonatal GBS, important disparities remain. While the incidence of early GBS infection has declined among all racial groups in the United States, early onset GBS continues to affect African American children disproportionately, even after adjustments for higher preterm delivery rates [32].

In spite of the progress made by intrapartum prophylaxis, GBS remains the leading cause of early neonatal morbidity and mortality [32]. As a result, researchers have been investigating maternal immunization against GBS as a potentially better strategy in the prevention of neonatal sepsis. Maternal immunization could reduce colonization rates, provide protection against overwhelming infection through transplacental antibody transfer, and ultimately preclude the need for universal screening and prophylaxis [32, 33]. For other infections, immunization has already proven to be an essential tool for prevention efforts.

## 3. Immunizations

Effective immunization programs prevent sepsis. Though the development of an immunization against GBS has proven challenging, immunization has successfully mitigated other causes of overwhelming infection. In fact, infections with *Haemophilus influenzae* type B (HIB) and *Streptococcus pneumoniae*, the leading causes of bacterial meningitis, pneumonia, and overwhelming infection in children are now virtually 100% preventable through immunization [34]. Immunization is such a powerful tool against sepsis that adoption of underutilized or new immunizations worldwide by national immunization programs has been deemed essential to any efforts to reduce child mortality [35]. The case of HIB immunization provides a clear example.

### 3.1. HIB: Preventing Sepsis through Immunization

HIB leads to at least 3 million cases of severe disease and nearly 400,000 deaths every year [36]. The most concerning manifestations of HIB infection, including pneumonia, meningitis, and other life-threatening invasive infections, occur principally in infants and children less than two years of age [36]. In the United States, children less than five years of age account for more than 85% of children who suffer from invasive HIB disease, and bacteremia represents the majority of invasive disease [37]. Though HIB disease occurs throughout the world, the burden of disease remains more predominantly in nations with poorer resources [36]. Typical invasive HIB diseases include not only bacteremia and meningitis but also pneumonia, epiglottitis, cellulitis, and osteoarticular infections [36, 38]. In unimmunized populations, invasive HIB remains a prominent cause of bacterial meningitis in infancy, and HIB meningitis results in death in up to 20% of infected persons in spite of appropriate antibiotic management [36]. HIB infection is a formidable disease with considerable mortality and morbidity, but primary prevention through immunization has proven effective.

The conjugate immunization against HIB has prevented morbidity and mortality from HIB infection so successfully that the production of the vaccine has been regarded as one of the significant public health advances of the last century [38]. Through direct and herd effects, immunization against HIB has changed the epidemiology of HIB disease. Before immunization against HIB, the nasopharynx of most nonimmune children was colonized with HIB. While only a small percentage of HIB carriers manifest clinical disease, HIB carriers are critical disseminators of the bacteria [36]. In populations with high rates of HIB immunization, nasopharyngeal colonization rates are considerably lower [36]. More importantly, research has shown that HIB immunization reduces substantially the incidence of HIB infection in both immunized people and in children too young to receive HIB immunization [38].

In fact, the sizeable burden of HIB disease worldwide is almost entirely preventable through immunization. Currently, most HIB-related morbidity and mortality occur in developing nations where invasive HIB disease remains a substantial public health concern and where population-wide HIB immunization programs have not yet been employed [36]. Immunization remains the most effective tool against invasive HIB disease in both developed and developing nations. Near elimination of serious HIB disease occurs within years in most nations that introduce HIB immunization into their national immunization programs [36]. As a result, the World Health Organization (WHO) recommends that the conjugate HIB vaccine should be included in all infant immunization programs with the first immunization occurring as soon after six weeks of age as possible [36].

The use of HIB immunization has expanded in the last ten years. In 2004, only thirteen low-income nations routinely utilized HIB immunization, but four years later, in 2008, 66 low-income nations had implemented routine HIB immunization [39]. Similarly, in 2000, the only areas with widespread implementation of HIB immunization were in Europe and the Americas, but by 2006, 108 nations, with more than 55% of the world's children, had employed HIB immunization [39]. Now, nearly two-thirds of all childhood HIB deaths occur in merely ten nations in Africa and Asia [39]. This expansion of HIB immunization has been achieved through work by organizations such as WHO and the GAVI Alliance that seek to expand supplies of HIB immunization, to reduce the cost of immunization, and to aid nations with the introduction of the immunization [35]. Despite the proven value of immunizations, the recommendations of organizations such as WHO, and the efforts of organizations such as the GAVI Alliance, many low-income and middle-income nations have not yet adopted routine HIB immunization [35]. Further expansion of HIB immunization could further decrease childhood morbidity and mortality worldwide.

HIB provides an enlightening example of the great utility of immunization. Yet, immunizations have proven to prevent sepsis and other serious invasive infections from organisms other than HIB. Two other vaccine-preventable infections that cause substantial morbidity and mortality include *Streptococcus pneumoniae* and *Neisseria meningitides *(meningococcus). We will visit briefly these two infections.

### 3.2. *Streptococcus pneumoniae*

*Streptococcus pneumoniae* is one of the primary causes of sepsis and other serious invasive diseases among young children and children in low-income nations [40]. It may be responsible for as many as one million childhood deaths every year [40]! Moreover, antibiotic resistance of pneumococci has been increasing, a reality that further inspires an increase in the use of prevention strategies [40]. Meanwhile, immunization with the pneumococcal conjugate vaccine (PCV) has been shown to prevent overwhelming pneumococcal infection. As a result, PCV is now recommended for use in routine immunization programs [40]. Of note, however, recent research has revealed that though the rate of hospitalization for pneumonia in children has decreased following the introduction of PCV, the prevalence of empyema, a concerning complication of a small percentage of pneumonia hospitalizations, has increased considerably since the start of routine PCV immunization [41]. Nevertheless, further expansion of this immunization could substantially reduce the incidence of overwhelming infection from *Streptococcus pneumoniae* and thereby reduce pediatric mortality and morbidity worldwide.

### 3.3. Meningococcus

WHO also recommends that national childhood immunization programs include the conjugate vaccine against *Neisseria meningitides*. Meningococcus is a bacteria associated with a high rate of morbidity and mortality due to the sudden onset and rapid progression of infection, particularly in adolescent populations [43]. Multiple meningococcal immunizations exist. In the United States, the CDC recommends immunization with the tetravalent conjugate meningococcal vaccine, and immunization of adolescents with this vaccine has become routine [44]. The meningitis belt of sub-Saharan Africa, however, has the highest rates of meningococcus meningitis worldwide. In this part of the world, Group A meningococcus accounts for the vast majority of cases and causes epidemics every 7 to 14 years [45]. WHO therefore advocates for the introduction of a new meningococcal A conjugate vaccine throughout more than two dozen nations in the African meningitis belt. This immunization became available in December 2010 [45]. High coverage of this immunization in children and young adults could eliminate meningococcal A from that region. As with HIB and *Streptococcus pneumoniae*, immunization has become a powerful tool in the prevention of overwhelming invasive infection from meningococcus.

### 3.4. Refusal to Immunize

Though immunizations have eliminated or reduced the incidence of many infectious diseases that cause substantial morbidity and mortality, parental refusal to immunize their children has become a relatively common occurrence in the United States. Parents often cite concerns around immunization safety for their refusal to immunize [46]. Because success of an immunization program requires high coverage rates, increasing refusal to immunize makes populations vulnerable to disease. In fact, geographic clustering of refusals has resulted in outbreaks of vaccine-preventable illnesses [47]. Any prevention effort that utilizes immunization must therefore recognize and manage this significant barrier to success.

## 4. Healthcare Associated Infections

A third strategy in the prevention of sepsis is the prevention of healthcare-associated infections (HAIs), an area of medicine that has received increasing amounts of attention in recent years. HAIs are infections that occur to patients while they are receiving care for other conditions, and they represent the most common complication affecting hospitalized patients today, with currently 5 to 10 percent of patients in acute care hospitals acquiring one or more infections [48]. Surgical site infections (SSI), central line-associated bloodstream infections (CLABSI), catheter-associated urinary tract infections (CA-UTI), and ventilator-associated pneumonia (VAP) account for the vast majority of all HAIs. These infections frequently lead to hospital-acquired sepsis and subsequent death. Approximately, 2 million patients experience HAIs every year in the United States, and these infections result in approximately 90,000 deaths. HAIs also account for an estimated $4.5 to $5.7 billion in additional costs every year [48]. Prevention of these infections has therefore become a major priority in healthcare. In 2005, the Institute for Healthcare Improvement launched its 100,000 Lives Campaign, which is now the 5 Million Lives Campaign, targeting prevention and reduction of SSIs, CLABSIs, and VAPs. As of October of 2008, the Center for Medicare and Medicaid Services (CMS) has stopped reimbursing hospitals for expenses related to the treatment of certain HAIs, including SSI, CA-BSI, VAP, and CA-UTI [49].

There is a growing body of evidence that the use of “bundles” can lower the rate of certain HAIs in both hospitalized children [50–53] and adults [54, 55]. While implementing any one of the specific interventions in a bundle may not significantly impact the overall rate of HAIs, implementing all of the elements in aggregate can dramatically lower the rates of these infections. For example, the Cincinnati Children's Hospital Medical Center CA-BSI insertion and maintenance bundles are shown in Table 2. Reliable and widespread implementation of these two bundles in all of the inpatient units at our hospital resulted in a dramatic reduction in the overall rate of CA-BSI [53, 56].

Prevention of HAIs is an important component in preventing sepsis. Multimodal interventions have proven successful at reducing HAI rates. Systematic application of evidence-based practices that aim to reduce HAIs is therefore an important and necessary part of any effort to prevent sepsis.

## 5. Conclusions

Given the substantial toll that sepsis takes worldwide, prevention of sepsis remains a global priority. Multiple effective prevention strategies exist. Antibiotic prophylaxis, immunizations, and healthcare quality improvement initiatives are important means through which we may reduce the morbidity and mortality from sepsis around the world. Inclusion of these strategies in a coordinated and thoughtful campaign to reduce the global burden of sepsis is necessary for the improvement of pediatric health worldwide.

## Figures and Tables

**Table 1:** Conditions for which antibiotic prophylaxis is recommended [42].

Prosthetic cardiac valve or prosthetic material used for cardiac valve repair

Previous infective endocarditis

Cardiac transplantation recipients who develop cardiac valvulopathy

Congenital heart disease
(i) Unrepaired cyanotic CHD, including palliative shunts and conduits
(ii) Completely repaired congenital heart defect with prosthetic material or device, whether placed by surgery or by catheter intervention, during the first 6 months after the procedure
(iii) Repaired CHD with residual defects at the site or adjacent to the site of a prosthetic patch or prosthetic device (which inhibit endothelialization)

**Table 2:** Key elements in the CCHMC CA-BSI insertion and maintenance bundles.

Insertion bundle
(1) Strict hand hygiene
(2) Full sterile barrier precautions
(a) Catheter insertion training for all credentialed providers
(b) Fully stocked insertion bin/cart
(c) Sterile gown, hat, mask, gloves worn by provider inserting the line
(d) Large sterile drape covering 80–100% of the patient and bed
(3) Chlorhexidine skin scrub at insertion site (2 minute scrub, 1 minute air-dry)
(4) Chlorhexidine-impregnated sponge placed at insertion site
(5) Insertion checklist
(6) Staff empowerment to stop procedure, if necessary

Maintenance bundle
(1) Strict hand hygiene
(a) Hand washing or use of an alcohol-based hand gel by all personnel prior to any catheter care
(b) Wear gloves for all catheter manipulations, medication administration, and so forth
(2) Catheter site care
(a) Change clear dressing every 7 days unless visibly soiled, loosened, or dampened
(b) Change sterile gauze dressing every 2 days unless visibly soiled, loosened, or dampened
(c) Chlorhexidine scrub to site with dressing changes (30 second scrub followed by 30-second air-dry)
(d) No iodine ointment at site
(e) Chlorhexidine-impregnated sponge placed at insertion site with every dressing change
(f) Prepackaged dressing change kit used for all dressing changes
(3) Catheter/hub/cap/tubing care
(a) Replace continuous administration sets no more frequently than every 72 hours (no less frequently than every 96 hours), unless visibly soiled or contaminated
(b) Replace intermittent administration sets every 24 hours or sooner if visibly soiled or contaminated
(c) Replace tubing used to administer blood, blood products, or lipids within 24 hours of initiating infusion
(d) Cap change every 7 days and within 24 hours of blood product administration (sooner if visibly soiled or contaminated)
(e) Prepackaged cap change kit
(4) Daily discussion of line necessity and integrity
